# Cervical spine clearance in pediatric trauma patients - Consensus algorithm of the Pediatric Spinal Trauma Group of the Spine Section of the German Society for Orthopedic and Trauma Surgery (DGOU)

**DOI:** 10.1007/s00068-026-03088-6

**Published:** 2026-06-16

**Authors:** Julia Bolte, Hauke Rüther, Pia Brecht, Dina Wilma Wiersbicki, Yasmin Youssef, Jan-Sven Jarvers, Alexander Carl Disch

**Affiliations:** 1 University Comprehensive Spine Center (UCSC), University Center for Orthopedics, Trauma and Plastic Surgery, Faculty of Medicine and University Hospital Carl Gustav Carus, TUD Dresden University of Technology, Dresden, Germany; 2 Department of Trauma Surgery, Orthopedics and Plastic Surgery, University Medical Center Goettingen, Goettingen, Germany; 3 Department of Pediatric Orthopedic and Trauma Surgery, Helios Klinikum Emil von Behring Berlin, Berlin, Germany; 4 Department of Orthopaedic, Trauma and Plastic Surgery, University of Leipzig Medical Center, Leipzig, Germany; 5 Department of Trauma and Orthopedic Surgery, Johann Kentmann Clinic Torgau, Torgau, Germany

**Keywords:** Pediatric spinal trauma, Pediatric cervical spine injuries, Imaging of the pediatric cervical spine, Cervical spine clearance in pediatric

## Abstract

**Purpose:**

Although cervical spine injuries in pediatric patients are rare, clinical and radiological examinations are clinical routine. Currently, no standardized decision algorithm does exist for detection of these injuries in the European region. The proposed algorithm is aiming to safely identify significant injuries through consistent diagnostics while minimizing immobilization and radiation exposure at the same time.

**Methods:**

The *Pediatric Spinal Trauma Group* of *the Spine Section of the German Society for Orthopedic and Trauma Surgery (DGOU)* developed an algorithm based on own scientific investigations and considering primary and secondary literature. The algorithm was developed through a formal consensus process and thereby aligns with international recommendations.

**Results:**

The algorithm uses the initial pediatric Glasgow Coma Scale (pGCS) to group patients to three treatment pathways. Resulting recommendations for imaging, clinical re-assessment, and further management include the child’s age, trauma mechanism, and clinical evaluations.

**Conclusion:**

Despite their rarity, potential cervical spine injury in children requires accurate diagnosis. Clinical examinations can be challenging, especially in children with compromised consciousness. Increased radiation exposure from X-rays and computer tomography (CT) scans must be considered, particularly as magnetic resonance imaging (MRI) availability is sometimes limited and often requires more resources as sedation or anesthesia in younger children. The introduced algorithm uses standardized pathways for the diagnosis of pediatric cervical spine injuries aiming to reduce the number of missed injuries as well as diagnostic delays, minimize unnecessary radiation exposure, and optimize healthcare resource utilization.

Spine Section of the German Society for Orthopedics and Trauma Surgery (DGOU)

**Supplementary information:**

The online version contains supplementary material available at 10.1007/s00068-026-03088-6.

## Introduction

Spinal injuries in children and adolescents are rarities, accounting for 1–4% of all fractures [3, 14, 21]. Alongside the thoracolumbar junction, the cervical spine is the most frequently affected region. The majority of cases occurs in children under the age of ten, predominantly located at the upper cervical spine [32]. Obtaining medical history often remains fragmentary and clinical examination can be challenging [30]. Despite daily presentation of potential pediatric cervical spine injuries in emergency departments, clear algorithms for examination and treatment are often lacking, even in experienced hospitals. A recent survey by our working group confirmed these findings [33]. Therefore, a simple and consistent diagnostic algorithm is essential to speed up and improve diagnostic and treatment procedures while reducing unnecessary immobilization as well as radiation exposure due to non-indicated imaging [1, 38]. This seems even more important, as magnetic resonance imaging (MRI) availability remains an issue even in international comparisons limited and associated protocols are inconsistent in regard to the literature [13, 24]. The Pediatric Cervical Spine Clearance Working Group (PCSCWG) published a consensus algorithm for the Anglo-American region in 2019 [15]. However, due to regional differences, an uncritical transfer of the recommendations to the European area does not appear to be expedient [18, 22]. The aim of the current publication is to present a standardized diagnostic algorithm for cervical spine clearance in pediatric trauma patients.

## Materials and Methods

### Working group

*The Pediatric Spinal Trauma Group* of *the Spine Section of the German Society for Orthopaedic and Trauma Surgery (DGOU)* is a panel of surgeons, that are actively involved in the care of pediatric spinal trauma. Members are affiliated up to supra-regional trauma centers and Deutsche Wirbelsäulen Gesellschaft (DWG)-certified spinal centers where treatment is carried out on an interdisciplinary basis with neurosurgeons. Furthermore the authors contributed to recent publications and guidelines on pediatric spinal trauma. The research revealed that only a small number of reliable studies have been published. Therefore, the algorithm was created based on the best available evidence. For the development of the algorithm “Cervical spine clearance in pediatric trauma patients”, the members of the working group participated in eight sessions to develop a consensus algorithm for the clinical and imaging diagnosis of potential pediatric cervical spine injuries in the European region using a nominal group process [2].

### Literature Review and Algorithm

A narrative review was conducted, analyzing available research findings and the working groups own published work on the diagnosis and treatment of pediatric spinal injuries. A search of primary and secondary literature on the subject of diagnosis and treatment of spinal injuries in children was carried out, including the keywords pediatric cervical spine injuries and trauma, cervical spine clarity algorithm, cervical spine immobilization, pediatric spine, and pediatric cervical spine fracture. Care was taken to refer mainly to reviews. An internal literature database of the working group on pediatric spinal injuries was established in 2016 and additionally included. This database contains around 60 publications from the German and Anglo-American language areas published between 1988 and 2018 [27, 40]. Previous achievements of the mentioned working-group include a retrospective multicenter study, publications of diagnostic and treatment recommendations, related quality measures using a national survey of surgeons, as well as the initiation of national guidelines [18, 27, 33, 40].

Based on the algorithm for the North American region by the PCSCWG, an adapted algorithm was developed for the Central European region [15].

Between September 2023 and February 2025, members of the above-mentioned working group participated in eight meetings for a formal consensus process based on existing international recommendations [2]. The first three meetings focused on the review the international evidence based and identify key differences in regional healthcare practices and classification of the literature, meetings 4–6 on the creation and revision of the algorithm aligning with existing international recommendations and considering the various resources available, and the last two meetings on the joint revision of the manuscript. Once consensus was achieved through the nominal group process, the findings were summarized into a compact and structured algorithm.

## Results

Emergency neurological status evaluation of pediatric patients usually follows the pediatric modified Glasgow Coma Scale (pGCS)[20]. As age is one of the most important factors in the clinical and radiological decision making process for identifying cervical spine injuries, imaging recommendations were given for the previously defined age groups. Due to similar protocols and previous publications the authors agreed to combine age groups 1 (0–6 years) and 2 (7–9 years) to one group, which was included into the imaging pathway [18, 27]. Age group 3 (10–16 years) thereby follows its own branch in the pathway.

### Initial pGCS 14–15 – Algorithm branch I

This diagnostic branch focusses on taking the medical history, as well as a detailed physical examination. Depending on the child’s age, medical history can be obtained directly from a patient, or via an accompanying person/witness. Depending on the results, a recommendation for imaging is given, referring to NEXUS-Criteria and Canadian-C-Spine-Rules [37].

**Fig. 1:**
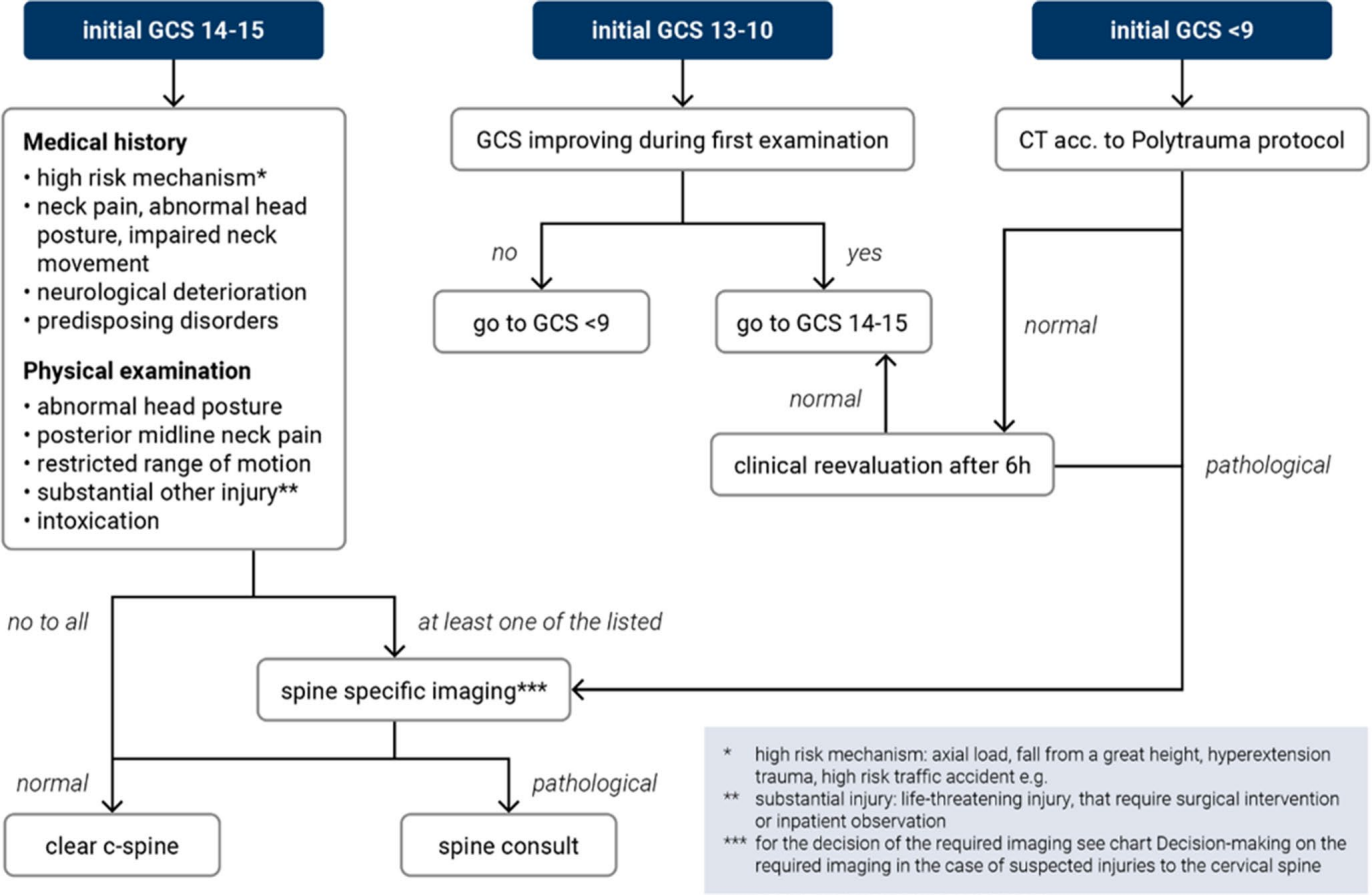
Algorithm for the treatment of childhood cervical spine injuries

If no high-risk trauma mechanisms, persistent neck pain, abnormal head posture, restricted neck movement, focal neurological deficits, or predisposing disorders of the cervical spine is reported and if physical examination shows no posterior midline pain, abnormal head posture, restricted range of motion of the cervical spine, inability of focus, or possible injury of chest, abdomen or pelvis, the cervical spine can be cleared (Fig. 1). If any of the above conditions apply, imaging must be considered.

### Initial pGCS 9–13 – Algorithm branch II

This group is known to be at intermediate risk for injury. Initial clinical clarification is not possible, and a follow-up examination is required. If the pGCS improves during first examination, children can be treated as described in algorithm branch I. If the pGCS does not improve, patients should be treated as described in algorithm branch III (Fig. 1).

### Initial pGCS < 9 – Algorithm branch III

Patients with an initial pGCS < 9 without improvement during initial examination should immediately undergo a computer tomography (CT) scan according to the current pediatric polytrauma guidelines. If the CT scan shows no pathologies, a clinical re-evaluation within 6 h is recommended. As long as a pathology has not been completely ruled out, the immobilization should be maintained. In the clinical re-evaluation, particular attention should be paid to a neurological deterioration. Since an active examination is significantly limited in this patient collective, special attention must be given to neurological pathologies such as pathological reflexes, or overflow incontinence. In case of a pGCS improvement, the patient can be further diagnosed according to algorithm branch I (pGCS 14–15). If the CT scan reveals pathological findings or is unremarkable but the pGCS does not improve, further imaging should be performed (Fig. 1).

### Imaging of the cervical spine

Children aged 0–9 years are grouped together regarding imaging of the cervical spine. In this age group, emergency MRI is required if cervical spinal trauma is combined with a focal neurological deficit. In the absence of neurological symptoms, MRI imaging can be downgraded to urgent. Children 10 years and older also require an emergent MRI if they do present with a trauma and a focal neurological deficit. If neurological symptoms are absent, and the initial pGCS is > 9, at least a lateral view X-ray of the cervical spine should be performed in this group.

X-ray imaging of the cervical spine is not considered as a sufficient diagnostic tool in patients under the age of 10 after trauma [9, 36].

As mentioned, CT scans are primarily chosen for imaging in severe injured children and usually included in corresponding pediatric polytrauma protocols.

Additional imaging, such as dynamic fluoroscopy, should only be performed by an experienced spine surgeon on pain-free children. The mentioned constellation is rare and should be well considered in limited indications due to higher radiation exposure.

**Fig. 2:**
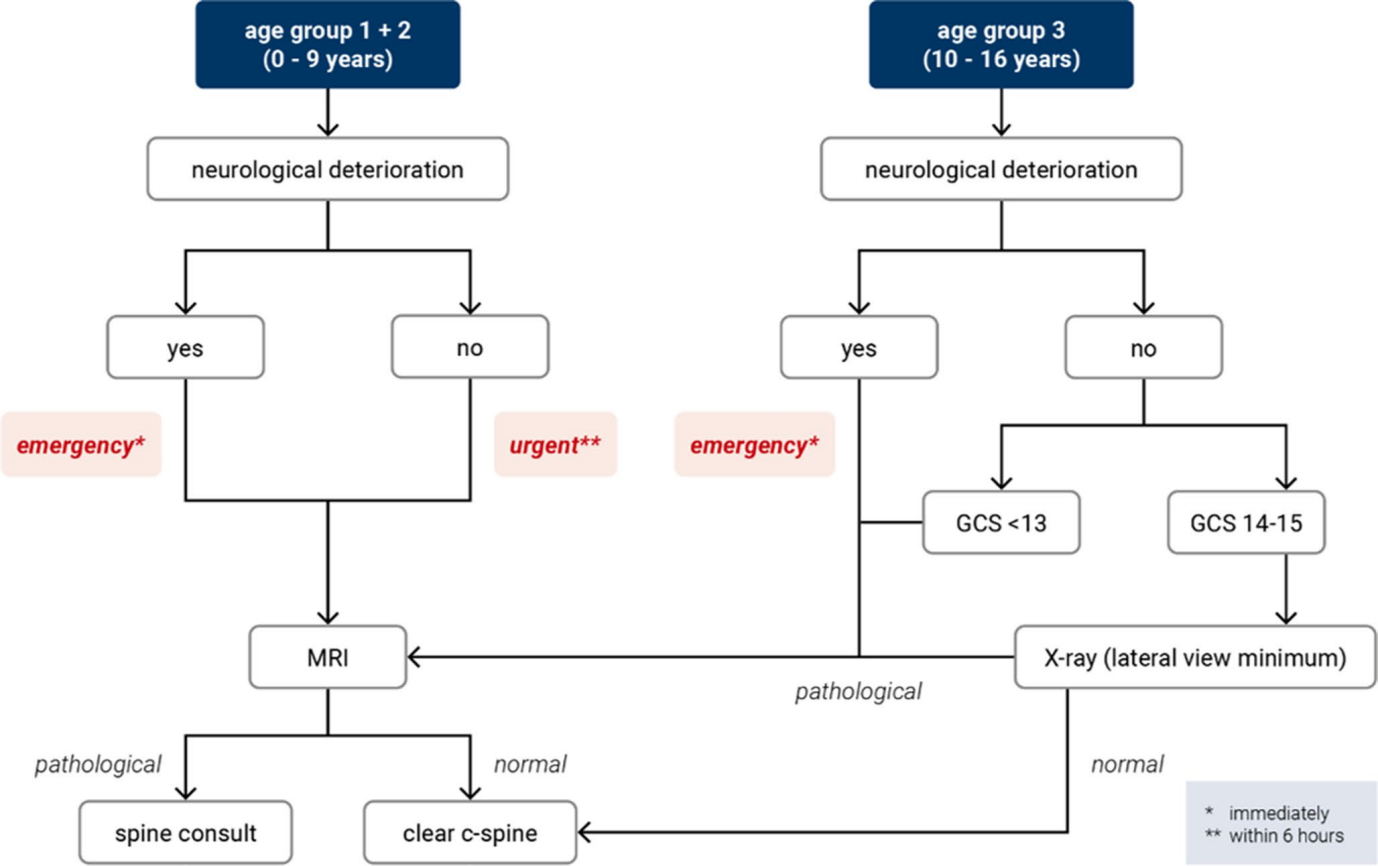
Decision-making on the required imaging in the case of suspected injuries to the cervical spine

In cases of diagnostic uncertainty or conceivable resource limitations, patients should be transferred to an experienced spine center with the possibility of an immediate MRI under immobilization of the cervical spine (Fig. 2).

## Discussion

### Pediatric Cervical Spine Clearance Protocols

Cervical spine injuries in children are rare, with an incidence of approximately 1–2% among polytraumatized children [29] and they make up to 1–4% of all pediatric fractures [3, 14, 21]. These injuries differ significantly in trauma mechanism, morphology, diagnostics and at least treatment from those in adults. Regardless of whether they present in the emergency room or during the consultation following a low-energy trauma. The high flexibility of the pediatric spine, varying size relations of head and spine, differing ligamentous biomechanical properties and relatively weak musculature often result in inadequate head and neck stabilization during high-velocity trauma [27].

Cervical spine clearance protocols in adult trauma patients have been shown to be effective in reducing missed injuries, minimizing radiation exposure and expediting cervical spine clearance. Furthermore, they lead to a reduction of overall socio-economic costs [7, 17]. According protocols for cervical spine clearance in children primarily exist and were lately developed in the Anglo-American region [25]. Hoffman et al. validated clinical criteria for adult trauma patients to reduce cervical spine radiographs [16]. Using the National Emergency X-Radiography Utilization Study (NEXUS) criteria, the authors reported 99% sensitivity in identifying 810 of 818 cervical spine injuries. In a secondary analysis of 3,065 patients under the age of 18 years concluded that the NEXUS criteria were also sufficient for children. Notably, only 30 children in this cohort were diagnosed with cervical spine injuries, with four cervical spine injuries in children < 9 years and none in children < 2 years [39]. Apart from that, the Canadian C-Spine rule (CCR), while well-known in adult patients [37], has not been validated in children. However, its modified use in pediatric guidelines and pediatric cohorts has been reported [6, 28, 35]. The validity of NEXUS in younger pediatric cohorts was questioned due to the low number of included children with cervical spine injuries [10, 11, 35]. Retrospective studies applying these criteria found that neither NEXUS nor CCR performs adequately for children under 8 years [11, 35]. More recently, the Pediatric Emergency Care Applied Research Network (PECARN) described eight variables associated with pediatric cervical spine injuries, which detected 98% of cervical spine injuries in their retrospective cohort while reducing imaging rates by up to 25% [24]. PECARN pilot work demonstrated the feasibility of developing a clinically sensible prediction rule. In a multicenter prospective observational study of 22,430 children aged 0–17 years presenting with blunt trauma at PECARN-affiliated centers, an accurate cervical spine injuries prediction rule was derived and validated. The rule achieved a sensitivity of 94.3%, specificity of 60.4%, and a negative predictive value of 99.9% [23]. This rule relied solely on clinical symptoms and physical examination findings, showing potential to reduce unnecessary CT scans without increasing plain X-ray usage.

Despite these advances, standardized recommendations for initial imaging in children are still lacking [29]. To provide such recommendations for the European region, the Pediatric Spinal Trauma Group of the Spine Section of the German Society for Orthopaedic and Trauma Surgery (DGOU) was established in April 2016. This group analyzed existing literature to formulate recommendations for diagnosing and treating pediatric cervical spine injuries [27]. Based on this work, a retrospective multicenter analysis of pediatric cervical spine injuries was conducted in six German spine centers [18]. It revealed, similar to previous studies, age-dependent injury patterns: upper cervical spine injuries were more common in children aged 0–9 years, whereas older children (over 9 years) more frequently sustained subaxial pathologies [18, 26].

Herman et al. developed uniform recommendations for the Anglo-American region, presented in a flowchart based on the Pediatric Glasgow Coma Scale [15]. The presented algorithm incorporates the initial pGCS assessment for further decision-making as previous studies do. To highlight the mentioned age-dependent differences in injuries, the algorithm has as second section, which contains age-specific recommendations for the selection of imaging (Fig. 2).

### Imaging

MRI holds significant importance in detecting cervical spine pathologies in younger children (≤ 9 years) (Fig. 2) [18, 26]. In this age group, the evaluation of the medical history and the physical examination can be challenging. Ossification of the cervical vertebral bodies is mostly completed by the age of 7–9 years, which can further limit the visualization of injuries on CT scans and X-Rays in younger children [8, 19]. CT radiation exposure raises lifetime cancer risks in children [4]. Therefore, balancing the risks of missed injuries against overuse of diagnostic imaging, particularly CT, is essential [15]. While plain radiographs are widely available, they remain controversial due to their lower sensitivity. A retrospective study found that 32% of 75 cervical spine injuries cases were missed using x-rays alone [12]. On the other hand, the main disadvantages of MRI imaging arises from limited availability, the prolonged scan times, the potential need for sedation, high costs, and false positive rates [15, 31]. In particular, with a significantly different care structure in Europe, an x-ray is much more available and can detect very straightforward injuries such as dislocations or subluxations. This information is important to differentiate between air or ground transport to a spinal center. As mentioned before, if the MRI is deemed necessary but is not available locally, the child must be transferred to a spinal center for further diagnosis.

Following previous recommendations [15], we suggest prioritizing initial CT imaging in cases of significant pGCS impairment (< 9), followed by MRI, according to common guideline recommendations [34]. This is in accordance to previous studies [5, 14]. Technological advancements may refine these recommendations, particularly with reduced CT radiation or faster MRI protocols. However, this resource is not immediately available in every hospital. Nevertheless, MRI remains the preferred diagnostic tool for younger patients. Plain radiographs are widely available and can play an important role in detecting primary injuries, particularly in older children (age group 3) with a pGCS of 14–15. In cases of diagnostic uncertainty, an MRI is recommended to confirm or rule out cervical spine injuries. If MRI is unavailable, at least a lateral X-Ray should be considered to avoid missing major instabilities.

### Limitations

There are several limitations in the development of this consensus algorithm. Cervical Pediatric spine injuries are rare, making it difficult to base recommendations on high-quality evidence. Due to low numbers, expert opinions were sometimes the highest achievable evidence during the Delphi process. Additionally, the project team was composed solely of orthopedic and pediatric orthopedic and trauma surgeons, introducing potential participant bias by absence of other professional societies. However, the authors participate in a multidisciplinary group that is working on a German guideline for pediatric spinal trauma.

The algorithm was primarily developed by physicians working in academic institutions, where clinical expertise and resources differ significantly from adult or smaller centers. As a result, full implementing of these recommendations may be challenging for smaller institutions. Moreover, the term “spine consult” is critical in each pathway, as expertise in pediatric spine injuries is not defined and can vary widely between institutions.

It should also be noted that the algorithm by Herman et al. [15] and the algorithm presented here show some similarities. However, the algorithm presented here is not only significantly simplified and supported by more recent data, but it also offers a separate action guideline for imaging, providing a clear and visually quicker recommendation for further radiological diagnostics. Further more this algorithm provides a clear time management which is crucial in the clinical decision making.

Finally, as noted by Herman et al. [15], further prospective studies are needed to assess the construct validity, specificity, sensitivity, and rate of false-negative findings. This algorithm needs to be validated by prospective studies to ensure its safety and applicability in everyday clinical practice in all pediatric age groups.

## Conclusion

This article presents an algorithm for cervical spine clearance based on the expert consensus statement by the Pediatric Spinal Trauma Group of the Spine Section of the German Society for Orthopaedic and Trauma using a modified Delphi method.

Based on the initial pGCS-Score 3 pathways were established and subdivided into age groups, aiming to maximize cervical spine injuries detection rate, reduce radiation exposure and optimize the use of available resources.

## Supplementary information

Below is the link to the electronic supplementary material.

Supplementary Material 1 (TIF 12.4 MB)

Supplementary Material 2 (TIF 12.4 MB)

## Data Availability

No datasets were generated or analysed during the current study.
