# Supplementary figures and images for: Cervical spine clearance in pediatric trauma patients - Consensus algorithm of the Pediatric Spinal Trauma Group of the Spine Section of the German Society for Orthopedic and Trauma Surgery (DGOU)
Source: Eur J Trauma Emerg Surg. 2026 Jun 16;52(1):196. doi: 10.1007/s00068-026-03088-6 (PMC13272246; doi:10.1007/s00068-026-03088-6)

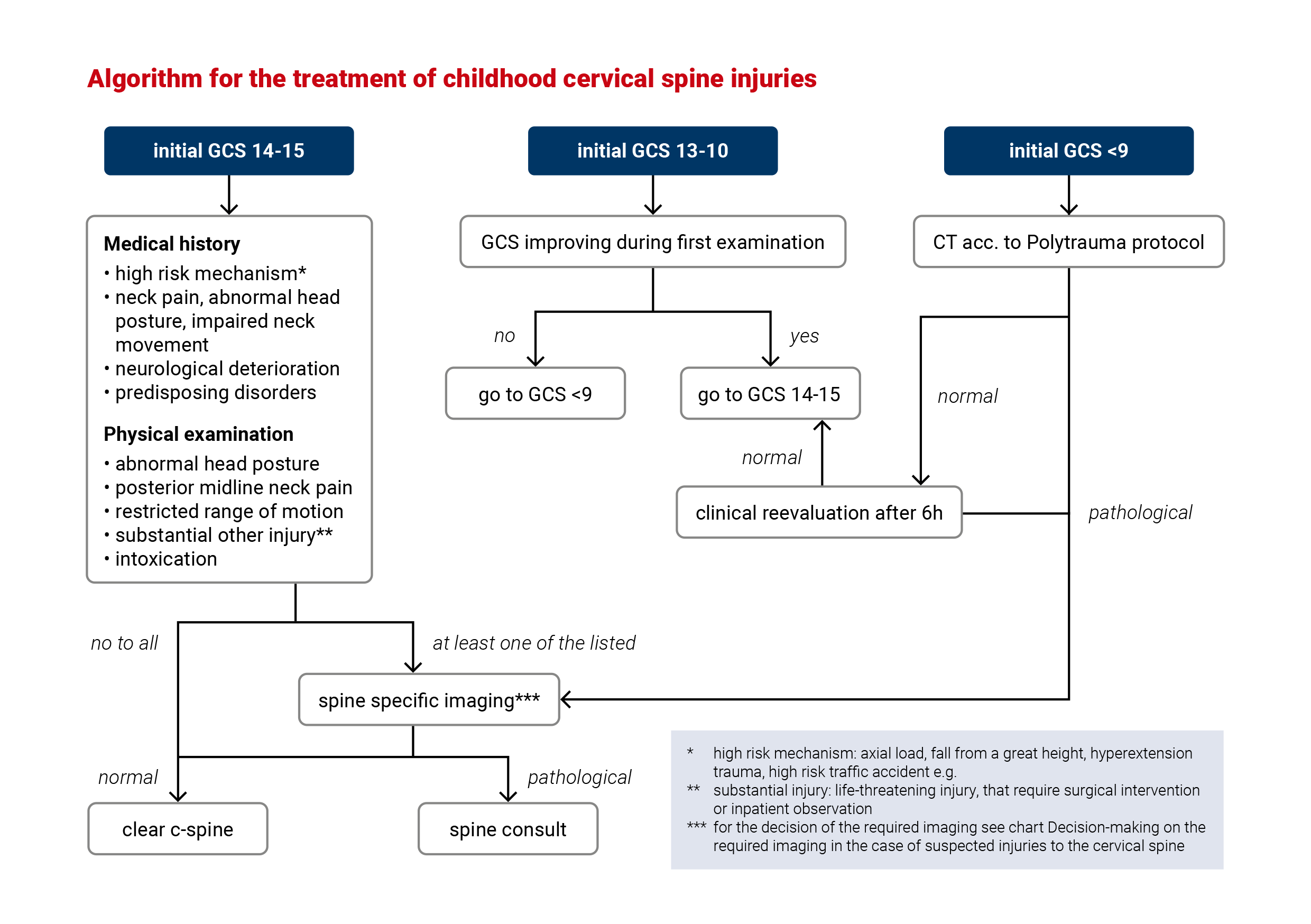

Supplement: Supplementary file 1 — Supplementary Material 1 (TIF 12.4 MB) [file 68_2026_3088_MOESM1_ESM.tif]

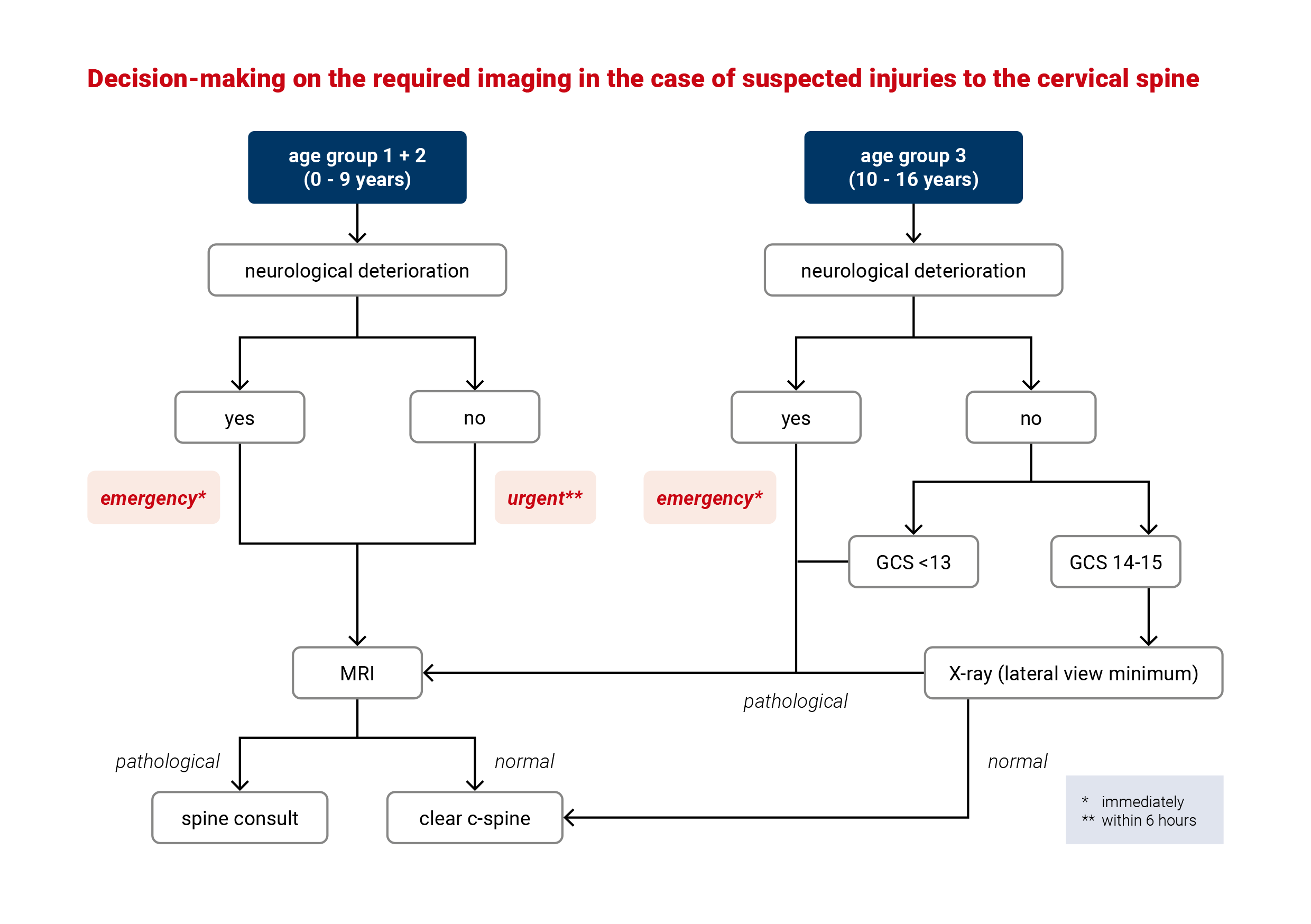

Supplement: Supplementary file 2 — Supplementary Material 2 (TIF 12.4 MB) [file 68_2026_3088_MOESM2_ESM.tif]
